# Health Impacts of Perchlorate and Pesticide Exposure: Protocol for Community-Engaged Research to Evaluate Environmental Toxicants in a US Border Community

**DOI:** 10.2196/15864

**Published:** 2021-08-11

**Authors:** Robert Trotter II, Julie Baldwin, Charles Loren Buck, Mark Remiker, Amanda Aguirre, Trudie Milner, Emma Torres, Frank Arthur von Hippel

**Affiliations:** 1 Department of Anthropology Northern Arizona University Flagstaff, AZ United States; 2 Center for Health Equity Research Northern Arizona University Flagstaff, AZ United States; 3 Department of Biological Sciences Northern Arizona University Flagstaff, AZ United States; 4 Regional Center for Border Health Inc. Yuma, AZ United States; 5 Yuma Regional Medical Center Yuma, AZ United States; 6 Campesinos Sin Fronteras Somerton, AZ United States; 7 Department of Community, Environment, and Policy Mel & Enid Zuckerman College of Public Health The University of Arizona Tucson, AZ United States

**Keywords:** community-engaged research, endocrine disruption, environmental contaminants, health disparities, toxic metal contamination, perchlorates, pesticides, population health, thyroid disease

## Abstract

**Background:**

The Northern Arizona University (NAU) Center for Health Equity Research (CHER) is conducting community-engaged health research involving “environmental scans” in Yuma County in collaboration with community health stakeholders, including the Yuma Regional Medical Center (YRMC), Regional Center for Border Health, Inc. (RCBH), Campesinos Sin Fronteras (CSF), Yuma County Public Health District, and government agencies and nongovernmental organizations (NGOs) working on border health issues. The purpose of these efforts is to address community-generated environmental health hazards identified through ongoing coalitions among NAU, and local health care and research institutions.

**Objective:**

We are undertaking joint community/university efforts to examine human exposures to perchlorate and agricultural pesticides. This project also includes the parallel development of a new animal model for investigating the mechanisms of toxicity following a “one health” approach. The ultimate goal of this community-engaged effort is to develop interventions to reduce exposures and health impacts of contaminants in Yuma populations.

**Methods:**

All participants completed the informed consent process, which included information on the purpose of the study, a request for access to health histories and medical records, and interviews. The interview included questions related to (1) demographics, (2) social determinants of health, (3) health screening, (4) occupational and environmental exposures to perchlorate and pesticides, and (5) access to health services. Each participant provided a hair sample for quantifying the metals used in pesticides, urine sample for perchlorate quantification, and blood sample for endocrine assays. Modeling will examine the relationships between the concentrations of contaminants and hormones, demographics and social determinants of health, and health status of the study population, including health markers known to be impacted by perchlorate and pesticides.

**Results:**

We recruited 323 adults residing in Yuma County during a 1-year pilot/feasibility study. Among these, 147 residents were patients from either YRMC or RCBH with a primary diagnosis of thyroid disease, including hyperthyroidism, hypothyroidism, thyroid cancer, or goiter. The remaining 176 participants were from the general population but with no history of thyroid disorder. The pilot study confirmed the feasibility of using the identified community-engaged protocol to recruit, consent, and collect data from a difficult-to-access, vulnerable population. The demographics of the pilot study population and positive feedback on the success of the community-engaged approach indicate that the project can be scaled up to a broader study with replicable population health findings.

**Conclusions:**

Using a community-engaged approach, the research protocol provided substantial evidence regarding the effectiveness of designing and implementing culturally relevant recruitment and dissemination processes that combine laboratory findings and public health information. Future findings will elucidate the mechanisms of toxicity and the population health effects of the contaminants of concern, as well as provide a new animal model to develop precision medicine capabilities for the population.

**International Registered Report Identifier (IRRID):**

DERR1-10.2196/15864

## Introduction

The Center for Health Equity Research (CHER) at Northern Arizona University (NAU) is conducting community-engaged health research in Yuma County, Arizona (Yuma, Somerton, San Luis, Rio Colorado). The primary community health stakeholders include the Yuma Regional Medical Center (YRMC), Regional Center for Border Health, Inc. (RCBH), Campesinos Sin Fronteras (CSF), Yuma County Public Health District, and several nongovernmental organizations (NGOs) working on border health issues. These stakeholders identified the most important regional priorities for joint health research [1]. One high-priority request was to examine basic epidemiology and conduct targeted translational research on environmental toxicants that impact communities in the border region.

Following discussions, literature reviews, and matching local needs with NAU research resources, we constructed this project as a joint community/university effort to examine the impact of human exposures to perchlorate, a water-soluble contaminant [2,3], and toxic metals such as cadmium, copper, lead, manganese, and mercury [4-11], which are active ingredients of currently or formerly used pesticides in the region. Results from the exposure assessment will be related to the health outcomes of Yuma residents. This project also includes the development of a new animal model for investigating the mechanisms of toxicity following a “one health” approach [12]. The ultimate purpose of this community-engaged effort is to develop interventions to reduce exposures and impacts of contaminants in Yuma populations.

In 2017, Yuma County had a population of 207,534 and an additional estimated 90,000 winter visitors/residents [13]. The race and ethnicity of the year-round population comprised 63.9% Hispanics, 30.8% White non-Hispanics, 2.7% African Americans, 2.3% Native Americans, 1.5% Asians, and 0.3% Native Hawaiians or other Pacific Islanders. The Yuma region sustains a large agricultural labor force [14] and frequent cross-border interactions with migrant farm workers from Mexico. In addition, Yuma County is home to two federally recognized tribes (Cocopah and Quechan). The United States Department of Labor’s Bureau of Labor Statistics ranked the unemployment rate of 387 metropolitan areas in November 2018 and found that Yuma had the second highest unemployment rate in the country, at 14.9% [15].

**Figure 1:**
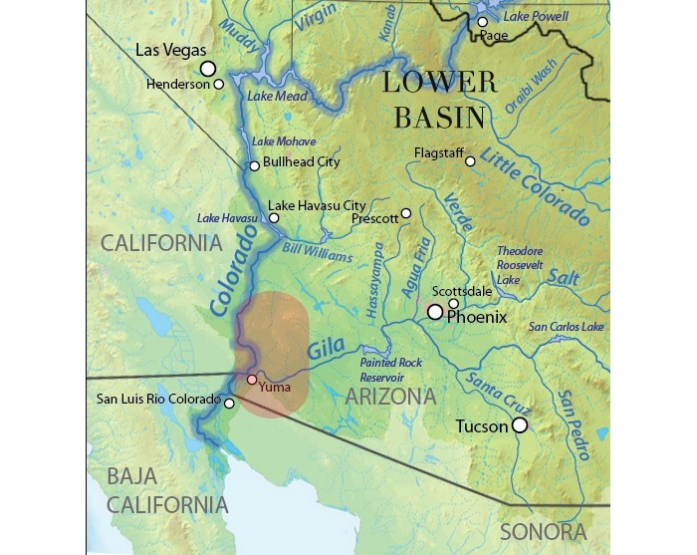
Map of the study region.

Yuma County is bounded by the Colorado River to the west and the US border with Mexico to the south (Figure 1). The Colorado River is the primary source of irrigation and drinking water throughout the region. The Colorado River, and thus the water used for irrigation and drinking, was contaminated with perchlorate that originated from a production facility in Nevada [16]. Perchlorate is a water-soluble and highly persistent environmental contaminant [17] that acts as an endocrine disruptor by outcompeting iodide at the sodium-iodide symporter of the thyroid gland, leading to hypothyroidism [18]. Perchlorate-induced hypothyroidism poses a particular risk during early development and has been linked to a significantly altered thyroid status in Yuma neonates [16]. In addition to affecting thyroid health, our research group discovered that perchlorate disrupts sexual development in laboratory animal models [19-23] and may therefore be a factor influencing the development of certain human reproductive problems. The animal model work also revealed that perchlorate can act as an obesogen, and therefore, it may play a role in the current obesity epidemic [24,25]. Because obesity in Hispanic immigrant populations is a high public health priority [26], this element of the proposed project may have strong implications for obesity-related programs.

Yuma County is often referred to as “the lettuce capital of the United States” and is a national source of winter vegetables (lettuce, cabbage, broccoli, kale, radish, and yellow squash), melons (cantaloupe, honeydew, and watermelon), citrus fruits (oranges and grapefruit), and dates. All these crops are a potential source of perchlorate exposure in the US food supply chain. The economy of the Yuma area is based upon year-round agriculture with intensive use of pesticides. Therefore, this project also focuses on exposure to toxic metals used in pesticides currently or in the past, such as mercury, lead, manganese, and copper. These metals are potent neurotoxicants when present at high concentrations and some also disrupt the endocrine system. Collectively, the population mix and environmental conditions in Yuma County provide a unique opportunity to investigate the health consequences of exposure to perchlorate, toxic metals, and pesticides.

In summary, Yuma is a medically underserved community that has historically experienced elevated exposure to perchlorate [16] and pesticides [27]; consequently, residents may face a higher-than-average risk of exposure but have poor access to resources and information to address this risk. The cultural diversity of Yuma County, combined with extensive intermediate-term residency (snowbirds) and the proximity of the United States–Mexico border, makes this a scientifically significant venue for implementing the protocol explained in this paper.

This pilot study was designed and conducted following a series of consultations with our community partners. We committed to examine the relationships between contaminant exposures and consequent health outcomes. We hypothesize that individuals with greater exposure to perchlorate will be more vulnerable to thyroid disorders, reproductive disorders, and obesity. Moreover, individuals with greater exposure to toxic metals have higher chances of experiencing neurological disorders. These hypotheses will be subsequently tested and published in later manuscripts. The current paper explains the feasibility of a community-engaged approach to field research in difficult-to-access populations and illustrates the success of the sampling methodology used to achieve our research aims.

## Methods

### Community-Approved Research Aims

The NAU investigators and the leadership from our community partners participated in a series of consultations about the purpose, design, and research protocols of this study. The study addresses community priorities regarding health equities for the Yuma region.

#### Community-Endorsed Aim

This pilot research protocol focused on comparing patients with thyroid disorders (n=147) that may be due, in part, to perchlorate exposure, with participants (n=176) having no known history of thyroid disease. Participants provided urine samples for the quantification of perchlorate, hair samples for the quantification of toxic metals, and blood samples for the quantification of thyroid and stress hormones. We will statistically model the associations between perchlorate and metal concentrations in individuals with their health outcomes, degree of endocrine disruption, and variables such as residency patterns, economic status, occupation, ethnicity, gender, and age.

### Chart Audits

The staff at the YRMC and RCBH reviewed the medical charts of patients to determine their eligibility for either the clinical group or the control group using a prescreening tool. The prescreening tool was designed to be culturally and linguistically appropriate by the research team in collaboration with community partners and was administered by the members of the research team. Participants were recruited based on the following eligibility requirements: over 18 years of age, year-round resident of the Yuma service region, and either experiencing a health problem under study (thyroid disease) or having no known history of thyroid diseases. A research team member then engaged with potential participants in either English or Spanish in the informed consent process. Participants voluntarily consented to participating in the research. Additional control participants from the community were recruited through CSF. The control participants had no record of thyroid disease. All participants were able to understand the informed consent process and the content of the survey.

### Recruitment

The recruitment, enrollment, and data collection process followed basic community-engaged research principles [28] and will follow these principles in terms of the analysis and dissemination of results at the individual, community, and scientific levels. Recruitment and data collection were accomplished by bilingual personnel from the YRMC, RCBH, and CSF. Survey questions were jointly vetted by community and university investigators, and feasibility measures were used to determine the possibility of scaling up the project.

Participant recruitment occurred at the following three sites: (1) YRMC: It is a not-for-profit health care system located in the city of Yuma, which is geographically centered between Phoenix and San Diego. The organization has 24/7 hospitalists and intensivists, more than 2000 employees, over 450 medical staff, and a family and community residency program accredited by the Accrediting Council for Graduate Medical Education (ACGME). The organization provides a comprehensive range of medical services at its main campus and facilities throughout the Yuma area. (2) RCBH: It has fully integrated behavioral and primary care rural health clinics in Somerton and San Luis, including an urgent care and a diagnostic medical facility. RCBH is the regional center for the Western Area Health Education Center, with offices in Yuma, La Paz, and Mohave Counties. RCBH also operates vocational technical schools called the “College of Health Careers” throughout its service area. (3) CSF: It was established in 1999 by a group of farmworkers who intended to address social, health, and environmental justice issues for migrant and seasonal farmworker families in Arizona. CSF is a 501(c)(3) not-for-profit, grassroots advocacy organization with a mission to promote self-sustainability for farmworker families, new immigrants, and low-to-moderate income individuals by providing and facilitating access to health care, behavioral health and social services, housing rehabilitation, counseling, immigration services, citizenship assistance, environmental education, and workforce development.

All participants completed the informed consent process in their language of choice. The informed consent included information on the purpose of the study, a request for access to participant health histories and medical records, and a brief survey. Following the informed consent process, in a single visit to the YRMC or RCBH, each participant was weighed and measured for BMI, and sampled for blood, urine, and hair. Whole blood (5 mL) was collected by venipuncture into a heparinized vacutainer and separated into plasma and cellular fractions via centrifugation; the plasma fraction was frozen and maintained at -80 ºC until assayed for hormone concentrations. Each participant also provided a single urine sample, which was stored at -20 ºC until analyzed for perchlorate concentration. Hair (~150 mg) was clipped close to the surface of the skin at the back of the neck using scissors and stored in paper envelopes at room temperature for later analysis of metals and metalloids. All sampling followed established quality assurance/quality control measures including the use of chain of custody and bio-banking forms.

### Survey Development

All consented participants completed a survey that included demographic data (age, gender, income, household composition), sources of drinking and cooking water, social determinants of health, a health status screen (eg, family history of diseases of the thyroid and reproductive organs), health care utilization information and access to care, and occupation and occupational exposure to environmental contaminants. This personal and population health information is being modeled with the measured levels of contaminants, endocrine function, and health status as determined from electronic medical records. Together, the findings will result in a clearer picture of the population health effects of contaminants and reveal the potential for developing precision medicine capabilities for the population.

### Electronic Medical Record Audit

For those individuals who consented to sharing their medical records, we transferred the data housed in NAU’s high-security server (in compliance with the Health Insurance Portability and Accountability Act [HIPAA] and National Institute of Standards and Technology [NIST]) through a Redcap (HIPAA-compliant) data transfer protocol. All data were de-identified prior to analyses, which were conducted behind the firewall of the secure information technology (IT) server.

Data from medical records were extracted through manual chart audits. The primary variables of interest comprised physical health diagnoses including thyroid conditions, cancers, obesity, diabetes, and hypertension; mental health diagnoses including anxiety, depression, sleep disorders, substance use disorders, and attention deficit disorders; medication history; and BMI.

## Results

**Figure 2:**
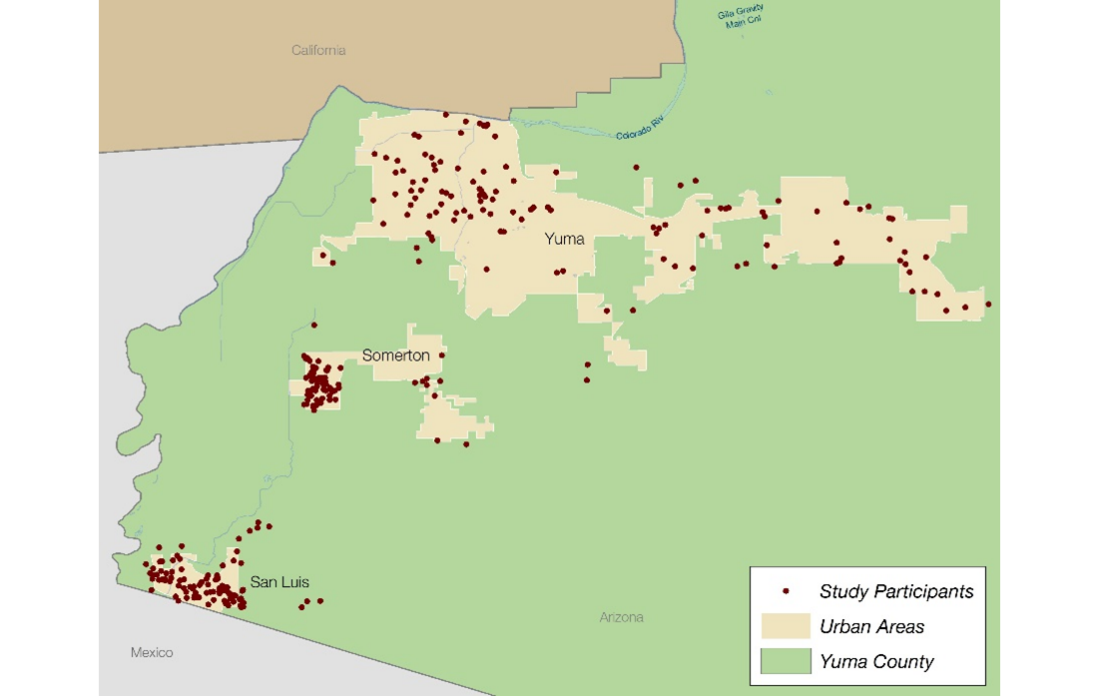
Geographic distribution of participant residences.

We determined the feasibility of our recruitment and data collection process. We recruited, consented, enrolled, and surveyed 323 adults residing in Yuma County (Figure 2). Among them, 147 residents were patients from either the YRMC or RCBH with a primary diagnosis of thyroid disease, including hyperthyroidism, hypothyroidism, thyroid cancer, or goiter. The remaining 176 participants were from the general population but with no history of thyroid disorder. We recruited 22 males and 123 females for the clinical sample, and 46 males and 132 females for the community sample. Most participants were Whites (251/323, 78%), and Hispanics or Latinos (286/323, 89%).

The participant demographics are presented in Tables 1 and 2. The clinical and community samples differed somewhat in terms of the percentage of male and female respondents, and the overall age profile of the samples, but were congruent in terms of the time of residence in the Yuma region, residence types, and household sizes. More women participated in the study than men (Table 1); nearly all participants had lived in Yuma County for over 5 years (Table 2), and participants in the clinical sample were older (mean 53.27; SD14.29) than participants in the community sample (mean 44.89; SD 15.25). Among the participants enrolled in the clinical sample (ie, those with documented thyroid disorders), over 50% (92/145) reported a diagnosis of hypothyroidism. Additionally, obesity (169/318, 53%), high cholesterol (132/323, 41%), hypertension (107/323, 33%), diabetes (87/323, 27%), depression (73/321, 23%), and anxiety (68/320, 21%) were the most frequently cited chronic conditions across all participants. Although most participants reported having health care insurance (281/323, 87%) and access to medical care (307/323, 95%), self-reported health statuses were more variable with approximately half of the participants indicating “fair” to “poor” health.

Although there were slight variations in the participants’ marital status (married=197/323, 61%) and residence type (own homes=266/323, 82%), the size of the household and number of children ranged between 1 to 22 and 0 to 11, respectively. Most participants had completed high school (191/323, 59%) with approximately one-third reporting a college or postgraduate degree (98/323, 30%).

Over half of the participants were currently employed (170/323, 53%), with 14% (46/323) reporting that they worked as a farmer, rancher, or agricultural worker in the last year and 17% (55/323) reporting that they were exposed to pesticides in the workplace. The most cited occupations included homemakers (ama de casa) (87/323, 27%), medical assistants (21/323, 8%), students (13/323, 4%), and farmworkers (11/323, 3%). Annual household incomes ranged from less than $5,000 to over $70,000 with most participants earning between $10,000 and $40,000.

**Table 1:** Clinical and community sample numbers by gender (N=323).

Sex	All, n (%)	Clinical, n (%)	Community, n (%)
Male	68 (21)	22 (15)	46 (26)
Female	255 (79)	123 (85)	132 (74)
Total	323	145	178

**Table 2:** Clinical and community sample numbers by residence time in Yuma County.

Sample	<6 months, n (%)	6 months to 1 year, n (%)	1 to 3 years, n (%)	3 to 5 years, n (%)	>5 years, n (%)
Community (N=178)	1 (0.6)	2 (1.1)	8 (4.5)	6 (3.4)	161 (90.4)
Clinical (N=145)	1 (0.7)	1 (0.7)	3 (2.1)	4 (2.8)	136 (93.8)

One key element of our protocol was to assess the feasibility of our community-engaged design and recruitment within the context of the need for systematic public health and population health data collection and analysis. We followed a community-engaged (modified community-based participatory research [CBPR]) logic model described by Belone et al [29] to increase our understanding of the factors that contribute to successful partnerships, including contexts, group dynamics/equitable partnerships, intervention, and research and outcomes. The full description and results of this community-engaged approach are forthcoming, but the key elements supporting the overall conclusion of successful engagement are summarized in Table 3, obtained from our ongoing evaluation of the protocol.

One of the primary areas of assessment was the overall partnership “health” measure provided by ongoing monitoring of the stability of the developed relationships and group dynamics focused on common goals. A critical area of process evaluation was monitoring community and researcher views on appropriate levels of collaborative research development (Table 3). The key elements that constituted the bulk of the process evaluation for the project were monitoring community and researcher views on appropriate levels of collaborative research development, especially in the areas of “context,” “group dynamics,” and “research processes,” as well as determining the level of satisfaction with the dissemination of findings and relevance of the primary outcomes for the research project from community and researcher perspectives. Although the overall dissemination is still in process for the partner and scientific communities, our assessment of the impact is consistently positive.

**Table 3:** Key elements of successful engagement with community partners.

Key element	Illustrative quotes from community partners
Context: This dimension focuses on factors that influence partnerships, including historical contexts of trust/mistrust between universities and communities, the salience of health issues to the community, and the capacity and readiness to engage in a project.	Community priorities and salience of health issues *–* *-* *We were interested because it discussed the environment and how it might be impacting you.* *- I was interested in participating because of how the findings could inform us about the health and well-being of our community.*
Group dynamics: This dimension focuses on relationships, the partnering process, and the importance of structural agreements among partners to assure community benefits. Benefits might include increased capacity in community leadership and in research performance.	Partnership length and trust – *- We* * had been working with members of the NAU*^a^ *team for years and knew them from involvement in the ABRC*^b^ *advisory board/steering committee.* Staff and care coordinators appreciated that one team member delivered the project introduction in Spanish. *Listening to each other and respecting each other, as equal partners and valuing the information that each brings makes a big difference.* Shared responsibility - The division of labor fell out naturally. CSF^c^, the advocacy organization for the farmworkers, primarily recruited farmworkers, whereas the hospitals (YRMC^d^, RCBH^e^) recruited patients and collected clinical data and samples; NAU researchers conducted the laboratory analytical work and statistical analyses.
Intervention/research: This dimension includes the extent to which community partners have a voice in terms of how their cultural norms and knowledge are integrated into the research in designing interventions, methods, or instruments or the extent of bidirectional translation, implementation, and dissemination.	Partnership synergy – *Really being open with each other, listening to each other and respecting each other as equal partners and valuing the information that each brings again that makes a big difference...throughout the years, I learned yes you guys know a lot about research and different things but you don't know about my community, you don't know about the things that we live on a daily basis, the challenges we face so that makes me an expert on my own issues and then I can speak up and be at the table speaking to you with the same level of authority.*
Outcomes: This dimension ranges from intermediate systems (i.e., policy and capacity changes, power relation changes, sustainability, and increased cultural renewal) to improved health and social justice outcomes.	Capacity to create desired community changes – *I learned so much about the process of research and how heavy metals and materials could affect the health of the community.* *This project has the potential to improve the health of individuals in the community. We will be able to apply the findings of this research in our work at the hospital.* *Knowing sources and causes can help prevent poor health. They need tools and choices. We need to empower individuals. If our community is exposed to things and they can do something to change it, we need to give them choices.*

^a^NAU: Northern Arizona University.

^b^ABRC: Arizona Biomedical Research Commission.

^c^CSF: Campesinos Sin Fronteras.

^d^YRMC: Yuma Regional Medical Center.

^e^RCBH: Regional Center for Border Health.

## Discussion

This pilot study lays the groundwork for future research designed to reduce contaminant exposures and health disparities of Yuma residents. Based on a community engagement model, we have committed to measuring the concentration of perchlorate in urine samples, measuring toxic metals in hair samples, quantifying a variety of hormones in blood samples, and comparing these findings with medical records and self-disclosure health surveys from each of the individuals recruited. We are statistically modeling the associations between the concentrations of the contaminants in individuals and their health outcomes, degree of endocrine disruption, and variables such as residency patterns, economic status, occupation, ethnicity, gender, and age. The ongoing collaboration with our community partners has allowed relatively rapid data collection with strong feasibility measures, as noted in our preliminary results described above. Establishing the levels of exposure to environmental toxicants in the Yuma region will allow us to examine the relationships between contaminant concentrations and adverse health outcomes. Developing a locally available animal model for testing the hypotheses related to contaminant concentrations and health outcomes will potentially lead to future translational studies and evidence-based public health policy development. Additionally, this pilot project was intended to improve research capacity in a community-engaged framework for border populations, and our process education measures support this endeavor.

## Abbreviations

ACGME: Accrediting Council for Graduate Medical Education
CBPR: community-based participatory research
CHER: Center for Health Equity Research
CSF: Campesinos Sin Fronteras
HIPAA: Health Insurance Portability and Accountability Act
IT: information technology
NAU: Northern Arizona University
NGOs: nongovernmental organizations
NIST: National Institute of Standards and Technology
RCBH: Regional Center for Border Health, Inc.
YRMC: Yuma Regional Medical Center
